# Plasma Biomarkers of Inflammation, Endothelial Function and Hemostasis in Cerebral Small Vessel Disease

**DOI:** 10.1159/000438494

**Published:** 2015-08-08

**Authors:** Stewart J Wiseman, Fergus N Doubal, Francesca M Chappell, Maria C Valdés-Hernández, Xi Wang, An Rumley, Gordon D.O Lowe, Martin S Dennis, Joanna M Wardlaw

**Affiliations:** ^a^Centre for Clinical Brain Sciences, University of Edinburgh, Glasgow, UK; ^b^Institute of Cardiovascular and Medical Sciences, Royal Infirmary, University of Glasgow, Glasgow, UK

**Keywords:** Biomarker, Endothelium, Inflammation, Stroke, Lacunar

## Abstract

**Background:**

The cause of lacunar ischemic stroke, a clinical feature of cerebral small vessel disease (SVD), is largely unknown. Inflammation and endothelial dysfunction have been implicated. Plasma biomarkers could provide mechanistic insights but current data are conflicting. White matter hyperintensities (WMHs) are an important imaging biomarker of SVD. It is unknown if plasma biomarkers add predictive capacity beyond age and vascular risk factors in explaining WMH.

**Methods:**

We prospectively recruited patients presenting with non-disabling ischemic stroke, classifying them clinically and with the help of MRI as lacunar or cortical. We measured biomarkers of inflammation, endothelial dysfunction and hemostasis for >1 month after stroke and compared biomarker levels between stroke subtypes. We quantitatively calculated WMH. We used multiple linear regression analysis to model WMH as a function of age, sex, hypertension and smoking (the baseline model). We fitted exploratory models using plasma biomarkers as predictor variables to assess model improvement over baseline.

**Results:**

We recruited 125 patients. The lacunar group (n = 65) had lower tissue plasminogen activator (t-PA) levels in unadjusted (7.39 vs. 8.59 ng/ml, p = 0.029) and adjusted (p = 0.035) analyses compared with the cortical group (n = 60). There were no significant differences in the other plasma biomarkers. The results for t-PA were consistent with an updated meta-analysis, although the effect remains non-significant (standardized mean difference −0.08 (95% CI −0.25 to 0.09)). The baseline regression model explained 29% of the variance in quantitative WMH (R^2^ 0.289). Inflammatory biomarkers showed minor improvement over baseline (R^2^ 0.291), but the other plasma biomarkers did not improve the baseline model.

**Conclusion:**

Plasma t-PA levels appear to differ between lacunar and cortical stroke subtypes, late after stroke, independent of age, sex and vascular risk factors and may reflect endothelial dysfunction. Except for a minor additional predictive effect of inflammatory markers, plasma biomarkers do not relate to WMH severity in this small stroke population.

## Introduction

The cause of lacunar ischemic stroke, a clinical feature of cerebral small vessel disease (SVD), is largely unknown although systemic inflammation, endothelial dysfunction, failure of the blood-brain barrier or occlusive microthrombus have been implicated [1,2,3,4,5].

Plasma biomarkers of inflammation, endothelial dysfunction and hemostasis may provide mechanistic insights, although if measured too soon after stroke they might simply reflect the acute effects of stroke rather than background pathway activity.

The MRI features of SVD include white matter hyperintensities (WMHs) and are associated with ischemic and hemorrhagic stroke and dementia [6]. WMH predict an increased risk of stroke [6] and are associated with poor functional outcomes following stroke [7]. Plasma biomarkers may predict outcome after stroke [8] and could have a role in the management of stroke patients [9].

The relationship between plasma biomarkers and imaging biomarkers of SVD is not fully understood. Systematic reviews [2,10,11] are impeded by between-study heterogeneity and they differ in their conclusions. Generally, plasma biomarkers are raised in lacunar stroke compared with non-stroke healthy controls (used in most studies) but differences here are unsurprising, especially in the acute phase of stroke. The situation is less clear when lacunar stroke is compared to other ischemic stroke subtypes. In a systematic review and meta-analysis [10], we found differences in levels of fibrinogen, D-dimer, von Willebrand factor (vWF) and interleukin-6 (IL-6) between lacunar and non-lacunar stroke, and no difference or conflicting evidence for other biomarkers.

In 2 large population studies of subjects without stroke, higher inflammatory biomarkers were independently associated with higher WMH volumes [1,12] but not in 3 other studies [13,14,15]. Biomarkers of endothelial activation were associated with WMH in a cross-sectional analysis [16] and with WMH progression [4]. Flow-mediated dilatation studies have shown the presence of endothelial dysfunction in lacunar stroke patients compared with non-stroke controls [17].

Prior stroke studies often take the plasma samples too early making it difficult to isolate underlying trends independent from an acute phase response. Few studies assessed a range of biomarkers simultaneously in one population.

The purpose of this study was (1) to determine if there were differences in levels of plasma biomarkers of (a) inflammation, (b) endothelial dysfunction or (c) hemostasis between lacunar and cortical stroke subtypes, well after the acute event, as representative of 3 potential SVD mechanisms, adjusted for age and major vascular risk factors; (2) to update our meta-analysis and place current findings into context and (3) to assess the association between the 3 plasma biomarker groups and WMH, irrespective of stroke subtype.

## Methods

Our definition of SVD is in accordance with the STRIVE neuroimaging reporting guidelines [18].

### Patients

We prospectively recruited patients, as consecutively as possible, who presented with ischemic stroke of lacunar or mild (i.e. non-disabling) cortical subtype seen at our hospital stroke service, as detailed previously [19]. Patients with cortical stroke acted as controls because they have many similar risk factors, medications and extent of damage due to the acute ischemic stroke to patients with lacunar stroke, thus controlling for potential confounders and allowing us to differentiate findings specific to SVD. We excluded patients with contraindications to MR, hemorrhagic stroke or severe stroke, that is, disabling total anterior circulation stroke. The study was approved by the local research ethics committee (2002/8/64), and all patients gave written informed consent.

### Patient Investigations

Patients were assessed at presentation by an experienced stroke physician and all underwent investigations as follows: brain imaging on a 1.5T research MR scanner with a standardized protocol (details available on request), carotid Doppler ultrasound and electrocardiogram. We recorded past medical histories including hypertension, diabetes, hypercholesterolemia and smoking, and measured the blood pressure and blood lipids as per the usual stroke patient assessment.

### Stroke Subtype

We assessed stroke severity with the National Institute for Health Stroke Scale (NIHSS) [20] (but did not use NIHSS as selection criteria) and classified the stroke clinical syndrome (lacunar or cortical) according to the Oxfordshire Community Stroke Project [21]. We defined ‘lacunar stroke’ as per the classical clinical lacunar syndromes (pure motor weakness or sensory loss or both in face and arm, arm and leg or all three, ataxic hemiparesis or clumsy hand dysarthria syndrome). We defined ‘mild cortical stroke’ as a maximum clinical deficit of either one of the following: weakness or sensory loss in the face, arm or leg, or loss of higher cerebral function (dysphasia or neglect), or weakness in more than one limb in the presence of loss of higher cerebral function (all in keeping with a partial anterior circulation stroke) or a homonymous hemianopia suggestive of occipital cortical infarct (in keeping with a cortical posterior circulation stroke).

We then assessed whether a recent infarct on MR was firstly present and secondly whether it was cortical or lacunar. We based the final stroke subtype classification on both the clinical and radiological classification. Where the clinical classification differed from the radiological classification, the radiological classification was used – using clinical criteria alone can result in misclassification of infarcts in up to 20% of cases [22]. Where an infarct on imaging was absent, an expert panel with all available information assigned the final stroke subtype.

### Plasma Biomarkers

All patients had their blood sampled after a minimum of 1 and maximum of 3 months following the stroke to avoid the acute phase. Samples were spun and frozen for batch analysis, blind to clinical data. We measured markers of inflammation (C-reactive protein (CRP) tumor necrosis factor-alpha (TNF) and IL-6), endothelial activation (vWF and intracellular adhesion molecule-1 (ICAM)) and thrombotic/fibrinolytic activity (fibrinogen, tissue plasminogen activator (t-PA) antigen and D-dimer). Intra- and inter-assay variation on biomarker testing was between 3.3 and 12.5% (online suppl. table 1; for all online suppl. material, see www.karger.com/doi/10.1159/000438494).

### Image Analysis

All scans were reviewed by a neuroradiologist for the index infarct and rated for SVD features using standardized scales [23,24,25] including the Fazekas scale for WMH. A quantitative, volumetric measure of WMH (in mm^3^) was also calculated, as described previously [26]. We corrected for head size by dividing the quantitative WMH load by the intracranial volume. We verified the correlation between the visual WMH rating and the quantitative value.

### Statistical Analysis

We assessed differences in patient demographics and plasma biomarkers between stroke subtypes using Student's t test, the Mann-Whitney U test and the chi-square test, as appropriate. Quantitative WMH and some plasma biomarkers (CRP, TNF, IL-6 and D-dimer) were not normally distributed; so we log transformed these data.

We explored the association between stroke subtypes and plasma biomarkers with multiple linear regression analysis which allowed us to control for age, sex and vascular risk factors.

We used multiple linear regression to assess the contribution of plasma biomarkers in explaining variance in quantitative WMH volume (n = 98, 27 scans were unavailable for WMH quantification), irrespective of stroke subtype. We repeated the modeling with visually rated WMH, for which the full data set was available (n = 125).

We used standardized units (mean = 0, SD = 1) for all plasma biomarkers in the regression models. The standardized data have no units and are on the same scale, so different biomarkers can be added together into summed variables which reduces the number of predictor variables to help avoid model over-fitting. We verified the correlation between the components of the summed variables. The summed variables were the following: inflammation (INF) (CRP + TNF + IL-6), endothelial dysfunction (END) (vWF + ICAM) and thrombosis (THR) (t-PA + D-dimer + fibrinogen).

We fitted a baseline model with quantitative WMH volume as the outcome measure and age, sex, hypertension and smoking status as the predictor variables. Patients with a past history of tobacco use were classified as non-smokers, if they were non-smokers at the time of stroke. Two patients (1 lacunar stroke and 1 cortical stroke) had missing data for smoking status. We fitted 4 further models: Model 1 (baseline + inflammation (INF)), Model 2 (baseline + endothelial dysfunction (END)), Model 3 (baseline + thrombosis/fibrinolysis (THR)) and Model 4 (baseline + INF + END + THR).

We compared each model for improvement over baseline. Model improvement was defined as a reduction in residual standard error (RSE) and increase in adjusted r-square (R^2^).

We checked for multicollinearity between predictor variables using variance inflation factor. We checked model assumptions as follows: independence, linearity, constancy of variance and normality in the residuals. Alpha level for significance was p < 0.05. All analyses were performed with the statistical programming language R version 3.0.1 (http://www.r-project.org/) [27].

### Meta-Analysis

We used the Review Manager 5 software (The Cochrane Collaboration) to update our prior meta-analysis [10], calculating the standardized mean difference (SMD) using the inverse variance method and a fixed effects model with 95% CIs.

## Results

We recruited 125 patients: 65 with lacunar stroke and 60 with cortical stroke. The mean age of the total cohort was 66.4 ± 11.4 years and the median NIHSS score was 1 (Q1-Q3 1-2). The median time from stroke onset to blood sampling was 54.4 (Q1-Q3 36-74) days. Patient characteristics and plasma biomarkers by stroke subtype are listed in table 1.

### Differences between Stroke Groups

The lacunar group had fewer men (39 vs. 51, p = 0.004), were younger (64 vs. 69 years, p = 0.015) and suffered less atrial fibrillation (2 vs. 9, p = 0.042) compared with the cortical group (table 1).

### Plasma Biomarker Association with Lacunar Stroke

The lacunar group had lower t-PA levels compared with the cortical group (7.39 vs. 8.59 ng/ml, p = 0.029) in unadjusted analyses (table 1) and after adjustment for age, sex, hypertension, smoking, diabetes and atrial fibrillation (p = 0.035; table 2). There were no differences in the other plasma biomarkers between lacunar stroke and cortical stroke whether adjusted or not (online suppl. tables 2–4).

To determine if the reduced t-PA was related to smoking, we repeated the analysis in non-smokers only (lacunar stroke, n = 40 vs. cortical stroke, n = 46). t-PA levels remained lower in lacunar stroke (online suppl. table 5). Although the difference became non-significant when adjusted for age, sex, hypertension and diabetes, the change was slight, the regression coefficients and 95% CIs were similar [28] being −1.33 (95% CI −2.53 to −0.13) vs. −1.37 (95% CI −2.84 to 0.09) (table 2 and online suppl. table 6, respectively) and may reflect the reduced sample.

### Meta-Analysis

**Fig. 1:**
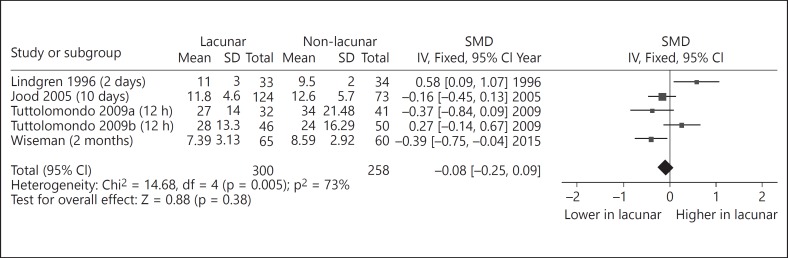
Forest plot comparing t-PA levels between lacunar stroke and non-lacunar stroke. Values in bracket after study refers to time to blood draw.

On addition of our study to the 4 prior studies (new total 300 lacunar strokes), we show lower t-PA in lacunar versus non-lacunar stroke although the difference was not significant (fig. 1). Addition of our data moves the SMD from 0.02 (95% CI −0.18 to 0.21) to −0.08 (95% CI −0.25 to 0.09).

### Biomarkers and WMH (All Patients: Lacunar and Cortical)

Quantitative WMH and plasma biomarkers were available for 98 patients. The baseline model (age, sex, hypertension and smoking status) explained 29% of the variance in quantitative WMH (RSE 1.081, R^2^ 0.289) with age, hypertension and smoking as significant predictors (table 3). Model 1 (baseline + INF) showed minor improvement over baseline (RSE 1.066, R^2^ 0.291). Models 2 (END), 3 (THR) and 4 (INF + END + THR) did not improve the baseline model. All models met model assumptions. There were no negative correlations between the components of the summed variables meaning a rise in one plasma marker was not offset by a fall in another (online suppl. table 7).

Visually rated WMH correlated strongly with quantitative WMH (r = 0.84, 95% CI 0.77 to 0.89). Models for visually rated WMH show similar results to quantitative WMH (online suppl. table 8).

## Discussion

We show a difference in t-PA levels between lacunar stroke and mild cortical stroke from plasma sampled well after the acute phase, independent of age, sex and risk factors. We did not find differences between stroke subtypes for biomarkers of inflammation (CRP, TNF and IL-6), endothelial dysfunction (vWF and ICAM) or other markers of hemostasis (fibrinogen and D-dimer). Except for a minor additional predictive effect of summed inflammatory markers, plasma biomarkers did not considerably improve the baseline model in explaining WMH.

### t-PA

t-PA is a glycoprotein released mainly by endothelial cells [29,30] to mediate the breakdown of thrombus. Its use as a thrombolytic agent might lead to the assumption that endogenous t-PA is protective against thrombosis [30]. However, higher t-PA antigen levels are associated with the risk of coronary heart disease in generally healthy populations [29]. This may reflect increased endothelial disturbance resulting in increased t-PA secretion or else increased levels of its inhibitor, t-PA inhibitor (PAI-1), resulting in increased levels of circulating complexes with t-PA [29,30,31]. Although smoking did not influence outcome after recombinant t-PA in IST-3 [32] some [33,34], but not all [35], observational studies suggest that smokers respond better to recombinant t-PA than non-smokers.

We found lower t-PA in those with lacunar as compared to those with cortical stroke. Reduced t-PA could mean lacunar stroke patients have reduced vascular damage – vWF levels were also lower in lacunar stroke but ICAM levels were higher (neither statistically significant). Alternatively, lacunar stroke patients might have increased endogenous fibrinolytic activity, if lower t-PA levels reflect lower levels of its inhibitor, PAI-1.

Knottnerus et al. [36] found significantly lower t-PA levels (and significantly higher PAI-1 levels) in 43 lacunar stroke patients with an isolated infarct versus 53 lacunar stroke patients with concurrent extensive WMH, hypothesizing that patients with extensive WMH lack the protective effect of PAI-1 in t-PA-induced tissue damage.

In our recent meta-analysis [10], t-PA was significantly higher in lacunar stroke patients than in non-stroke controls but did not differ significantly between patients of lacunar stroke and other stroke subtypes, although data are limited and the timing of sample collection could be confounding. Our samples were collected well after the acute phase and are more likely to reflect underlying pathway activity. The updated meta-analysis, including the current data, moves the evidence in favor of lower t-PA in lacunar stroke rather than non-lacunar stroke (fig. 1). The largest study to date to find lower levels of t-PA in small vessel stroke is the Sahlgrenska cohort, Sweden [37]: among 600 patients with ischemic stroke, including 124 with small vessel stroke, small vessel stroke patients had higher t-PA levels compared to non-stroke controls in the acute phase and at 3 months, but lower t-PA levels compared to patients with other stroke subtypes. The reduced t-PA is consistent with the impaired blood-brain barrier function found in the same cohort previously [38].

The lacunar group were significantly younger with fewer cases of atrial fibrillation and more smokers (non-significant) than the cortical group, although the association of lacunar stroke with lower t-PA was independent of these, and the pattern persisted in analysis restricted to non-smokers.

### WMH and Biomarkers

Age, hypertension and smoking were significant predictors of WMH. The inflammatory biomarker summed variable appeared to improve the model (slight reduction in the RSE) but the additional explanatory power was small and could be interpreted as no model improvement. On the other hand, a similar effect size confirmed in a larger study would indicate a modest but important effect of plasma markers of inflammation on WMH prediction. The other plasma markers did not have any additional explanatory power. Studies that have measured WMH in non-stroke populations typically involve older people. We have clearly shown age to be the most important predictor variable in the assessment of WMH and thus correcting for age is crucial.

Two large studies [1,12] showed independent associations between higher plasma inflammatory biomarkers and more WMH but had wide age ranges. However, Rouhl et al. [39] found no difference in CRP levels between 81 patients with and 265 patients without extensive WMH, Wersching et al. [13] found no association between CRP and WMH among 321 older stroke-free participants, Baune et al. [40] found no association between TNF and WMH among 268 community-dwelling participants and Aribisala et al. [41] found no association between inflammation (a latent factor comprising CRP, fibrinogen and IL-6) and WMH among 634 community-dwelling older people of near-identical age. Thus, it is possible that wide age ranges in some studies inflated associations between inflammatory markers and WMH. Shoamanesh et al. [15] found no association between some inflammatory biomarkers (including CRP, IL-6 and TNF) and SVD (defined as presence of silent infarcts and/or extensive WMH) in a large cohort of younger stroke-free Framingham participants (n = 522; mean age 60 years) but did associate ICAM with SVD. We found no association between ICAM and WMH in the present study but have much less power than the Framingham study. Our systematic review and meta-analysis [10] found no difference in ICAM levels between lacunar stroke and other stroke subtypes although only a few studies contributed data. ICAM was non-significantly higher in lacunar stroke patients than cortical stroke patients in the present study.

## Conclusion

Despite being small (n = 125) and cross-sectional, our study uses thorough methods and adds new information. Our findings show a difference in t-PA levels between lacunar and cortical stroke which should be verified in other data sets. Future studies should obtain plasma samples in the chronic phase after stroke and concentrate on longitudinal associations, especially the role of t-PA in stroke subtypes as it could help explain mechanisms. A large prospective study of accurately phenotyped stroke patients would be helpful. It is important to not only control for age specifically, but also hypertension and smoking when modeling features of SVD such as WMH and biomarkers.

## Funding

This study was supported by the Chief Scientist Office of the Scottish Government (CZB/4/281) (J.M.W. and M.S.D.), the Wellcome Trust (075611) (F.N.D. and J.M.W.) and a Principal's Career Development PhD Scholarship from the University of Edinburgh (S.J.W.).

## Disclosure Statement

The authors have no conflicts of interest.

## Supplementary Material

Supplementary data

## Figures and Tables

**Table 1:** Comparing patient characteristics and plasma biomarkers between lacunar and cortical stroke

	Lacunar stroke (n = 65)	Cortical stroke (n = 60)	p value
Male, n (%)	39 (60)	51 (85)	0.004^*^
Age, years, mean (SD)	64.1 (11.4)	69.0 (10.9)	0.015^*^
Hypertension, n (%)	37 (57)	39 (65)	0.458
Diabetes, n (%)	14 (21.5)	5 (8.3)	0.071
Current smoker, n (%)	24/64 (37.5)	13/59 (22.0)	0.095
NIHSS, median (Q1–Q3)	2 (1–3)	1 (0.75–2)	0.404
Time to sample, days, median (Q1–Q3)	56 (38–74)	52 (36–77)	0.894
Ischemic heart disease, n (%)	8 (12.3)	16 (26.6)	0.070
Atrial fibrillation, n (%)	2 (3.1)	9 (15)	0.042^*^
Hyperlipidemia, n (%)	26/64 (40.6)	23 (38.3)	0.939
Total cholesterol, mmol/l, mean (SD)	5.07 (1.10) (n = 57)	5.06 (1.13) (n = 53)	0.949
Positive family history of stroke, n (%)	10/64 (15.6)	4/58 (6.9)	0.220
Inflammation, median (Q1–Q3)			
CRP, mg/l	1.37 (0.84–3.44)	1.73 (0.97–3.54)	0.748
TNF, pg/ml	0.92 (0.72–1.33)	0.88 (0.76–1.23)	0.972
IL-6, pg/ml	2.57 (1.91–4.12) (n = 64)	2.58 (1.90–3.77)	0.994
Endothelial dysfunction, mean (SD)			
ICAM, ng/ml	162.78 (57.97)	159.27 (46.1) (n = 56)	0.711
vWF, IU/dl	129.31 (41.49)	131.7 (39.2)	0.741
Thrombosis/fibrinolysis			
Fibrinogen, g/l, mean (SD)	3.84 (0.61) (n = 64)	3.93 (0.67) (n = 59)	0.452
t-PA, ng/ml, mean (SD)	7.39 (3.13)	8.59 (2.92)	0.029^*^
D-dimer, ng/ml, median (Q1–Q3)	100 (73–157)	128.5 (73.25–182.5)	0.498

* p < 0.05.

**Table 2:** Association of t-PA with lacunar stroke subtype (n = 125)

	Regression coefficient (95% CI)	p value
Lacunar stroke subtype	–1.312 (–2.531 to −0.093)	0.035^*^
Age	–0.017 (–0.073 to 0.038)	0.530
Male sex	0.542 (–0.740 to 1.824)	0.404
Hypertension	0.393 (–0.806 to 1.593)	0.517
Smoking	1.027 (–0.277 to 2.333)	0.121
Diabetes	0.266 (–1.324 to 1.855)	0.741
Atrial fibrillation	0.240 (–1.854 to 2.334)	0.820

* p < 0.05.

**Table 3:** Explaining variance in quantitative WMH with different predictor variables (n = 98)

Predictor variables	RSE	R^2^
Baseline model		
Age^††^, male sex, hypertension^*^, smoking^†^	1.081	0.289
Model 1		
Age^††^, male sex, hypertension^*^, smoking^†^ + inflammation	**1.066**	**0.291**
Model 2		
Age^††^, male sex, hypertension^*^, smoking^†^ + endothelial activation	1.098	0.285
Model 3		
Age^††^, male sex, hypertension^*^, smoking^†^ + thrombosis	1.098	0.278
Model 4		
Age^††^, male sex, hypertension^*^, smoking^†^ + inflammation + endothelial activation + thrombosis	1.094	0.285

Inflammation = log CRP, log TNF, log IL-6. Endothelial activation = vWF, ICAM. Thrombosis = t-PA, log D-dimer, fibrinogen. For RSE, bold indicates improvement over baseline, i.e. reduction in RSE. For R^2^, bold indicates improvement over baseline, i.e. in-crease in R^2.^

* p < 0.05

† p < 0.01

†† p < 0.001.
