# Supplementary material for: Plasma Biomarkers of Inflammation, Endothelial Function and Hemostasis in Cerebral Small Vessel Disease
Source: Cerebrovasc Dis. 2015 Aug 8;40(3-4):157–64. doi: 10.1159/000438494 (PMC4611856; doi:10.1159/000438494)
Supplement: Supplementary file 1 — Supplementary data [file ced-0040-0157-s01.doc]

**ONLINE SUPPLEMENT**

**Title:**

Plasma biomarkers of inflammation, endothelial function and haemostasis in cerebral small vessel disease.

**Authors:**

*Stewart J. Wiseman^a^, *Fergus N. Doubal^a^, Francesca M. Chappell^a^, Maria C. Valdés-Hernández^a^, Xin Wang^a^, Ann Rumley^b^, Gordon D.O. Lowe^b^, Martin S. Dennis^a^, Joanna M. Wardlaw^a^.

* Joint first

^a^ Centre for Clinical Brain Sciences, University of Edinburgh, UK, and

^b^ Institute of Cardiovascular and Medical Sciences, Royal Infirmary, University of Glasgow, UK

**Tables:**

Supplementary Table 1. Assay details for plasma biomarkers

Supplementary Table 2. Association of plasma biomarkers of inflammation (CRP, TNF and IL-6) with lacunar stroke subtype (n=125)

Supplementary Table 3. Association of plasma biomarkers of endothelial dysfunction (ICAM and vWF) with lacunar stroke subtype (n=125)

Supplementary Table 4. Association of plasma biomarkers of thrombosis (fibrinogen and D-dimer) with lacunar stroke subtype (n=125)

Supplementary Table 5. Comparing patient characteristics and plasma biomarkers between lacunar and cortical stroke in non-smokers

Supplementary Table 6. Association of t-PA with lacunar stroke subtype in non-smokers (n=86)

Supplementary Table 7. Correlations between plasma biomarkers used in linear regressions (n=98)

Supplementary Table 8. Explaining variance in visually rated WMH with different predictor variables (n=125)

***Supplementary Table 1. Assay details for plasma biomarkers***

| **Blood markers** | **Measurement method** | **Unit** | **Intra-assay CV** | **Inter-assay CV** |
| --- | --- | --- | --- | --- |
| **Inflammation** |  |  |  |  |
| IL-6 | ELISA (R&D Systems, Abingdon, UK) | pg/ml | 7.5% | 8.9% |
| TNF | ELISA (R&D Systems, Abingdon, UK) | pg/ml | 8.4% | 12.5% |
| CRP | Immunonephelometry  (Prospec, Dade Behring Milton Keynes, UK) | mg/l | 4.7% | 8.3% |
| **Endothelial function** | | | | |
| vWF | ELISA (DAKO, High Wycombe, UK) | IU/dL | 3.3% | 4.2% |
| ICAM | ELISA (R&D Systems, Abingdon, UK) | ng/ml | 3.6% | 7.4% |
| **Thrombosis/fibrinolysis** |  |  |  |  |
| Fibrinogen | Immunonephelometry  (Prospec, Dade Behring Milton Keynes, UK) | g/l | 7.5% | 8.9% |
| t-PA | ELISA (Biopool AB, Umea Sweden) | ng/ml | 6.6% | 6.5% |
| D-dimer | ELISA (Biopool AB, Umea Sweden) | ng/ml | 4.7% | 5.2% |

**ELISA: enzyme-linked immunosorbent assay, CV: coefficients of variation, vWF: von Willebrand factor, ICAM: intracellular adhesion molecule, IL-6: interleukin-6, TNF: tumor necrosis factor alpha, CRP: C-reactive protein, t-PA: tissue plasminogen activator.**

***Supplementary Table 2. Association of plasma biomarkers of inflammation (CRP, TNF and IL-6) with lacunar stroke subtype (n=125)***

|  | **Regression coefficient (95% CI)** | **p value** |
| --- | --- | --- |
| **CRP** |  |  |
| Lacunar stroke subtype | -1.441 (-4.711 to 1.830) | 0.385 |
| Age | -0.024 (-0.173 to 0.126) | 0.753 |
| Male sex | 1.000 (-2.533 to 4.533) | 0.576 |
| Hypertension | 1.004 (-2.390 to 4.399) | 0.559 |
| Smoking | -0.182 (-3.760 to 3.396) | 0.920 |
| AVR | 0.490 (-1.065 to 2.046) | 0.534 |
|  | | |
| **TNF** |  |  |
| Lacunar stroke subtype | 0.288 (-0.203 to 0.779) | 0.247 |
| Age | -0.008 (-0.030 to 0.014) | 0.473 |
| Male sex | 0.004 (-0.526 to 0.534) | 0.988 |
| Hypertension | -0.162 (-0.671 to 0.347) | 0.530 |
| Smoking | -0.445 (-0.982 to 0.092) | 0.103 |
| AVR | -0.071 (-0.304 to 0.162) | 0.549 |
|  | | |
| **IL-6** |  |  |
| Lacunar stroke subtype | 0.366 (-0.423 to 1.155) | 0.360 |
| Age | 0.029 (-0.007 to 0.065) | 0.114 |
| Male sex | 0.365 (-0.486 to 1.217) | 0.397 |
| Hypertension | -0.068 (-0.891 to 0.754) | 0.869 |
| Smoking | 0.425 (-0.439 to 1.290) | 0.332 |
| AVR | 0.083 (-0.293 to 0.460) | 0.662 |
| ***AVR (additional vascular risk)*** = diabetes, hyperlipidaemia, atrial fibrillation, ischaemic heart disease, peripheral vascular disease, family history of stroke | | |

***Supplementary Table 3. Association of plasma biomarkers of endothelial dysfunction (ICAM and vWF) with lacunar stroke subtype (n=125)***

|  | **Regression coefficient** | **p value** |
| --- | --- | --- |
| **ICAM** |  |  |
| Lacunar stroke subtype | 2.552 (-18.420 to 23.524) | 0.810 |
| Age | -0.193 (-1.144 to 0.757) | 0.688 |
| Male sex | 7.954 (-14.608 to 30.517) | 0.486 |
| Hypertension | -3.688 (-25.350 to 17.974) | 0.737 |
| Smoking | 11.386 (-11.437 to 34.210) | 0.325 |
| AVR | -4.883 (-14.944 to 5.178) | 0.338 |
|  | | |
| **vWF** |  |  |
| Lacunar stroke subtype | 5.935 (-9.076 to 20.946) | 0.435 |
| Age | 1.225 (0.537 to 1.912) | 0.000 *** |
| Male sex | 5.275 (-10.937 to 21.488) | 0.520 |
| Hypertension | -3.808 (-19.386 to 11.770) | 0.629 |
| Smoking | 1.331 (-15.089 to 17.752) | 0.873 |
| AVR | 2.607 (-4.532 to 9.747) | 0.471 |
| *** p<0.001. ***AVR (additional vascular risk)*** = diabetes, hyperlipidaemia, atrial fibrillation, ischaemic heart disease, peripheral vascular disease, family history of stroke. | | |

***Supplementary Table 4. Association of plasma biomarkers of thrombosis (fibrinogen and D-dimer) with lacunar stroke subtype (n=125)***

|  | **Regression coefficient** | **p value** |
| --- | --- | --- |
| **Fibrinogen** |  |  |
| Lacunar stroke subtype | -0.108 (-0.351 to 0.135) | 0.382 |
| Age | 0.006 (-0.004 to 0.017) | 0.238 |
| Male sex | -0.000 (-0.262 to 0.262) | 0.998 |
| Hypertension | 0.063 (-0.189 to 0.317) | 0.620 |
| Smoking | 0.440 (0.174 to 0.706) | 0.001 *** |
| AVR | 0.006 (-0.109 to 0.122) | 0.908 |
|  | | |
| **D-dimer** |  |  |
| Lacunar stroke subtype | -22.986 (-90.559 to 44.587) | 0.502 |
| Age | 2.094 (-1.000 to 5.188) | 0.183 |
| Male sex | 12.475 (-60.505 to 85.456) | 0.736 |
| Hypertension | 5.056 (-65.070 to 75.182) | 0.887 |
| Smoking | 56.899 (-17.019 to 130.817) | 0.130 |
| AVR | -2.488 (-34.630 to 29.653) | 0.878 |
| *** p<0.001. ***AVR (additional vascular risk)*** = diabetes, hyperlipidaemia, atrial fibrillation, ischaemic heart disease, peripheral vascular disease, family history of stroke. For t-PA, see main text and Table 3. | | |

***Supplementary Table 5. Comparing patient characteristics and plasma biomarkers between lacunar and cortical stroke in non-smokers***

|  | Lacunar stroke (n=40) | Cortical stroke (n=46) | p value |
| --- | --- | --- | --- |
| Male sex, n (%) | 21 (52.5%) | 39 (84.7%) | 0.002 ** |
| Age, mean (SD) years | 66.5 (11.3) | 71.1 (10.9) | 0.055 |
| Hypertension, n (%) | 25 (63%) | 29 (63%) | 1.000 |
| Diabetes, n (%) | 9 (22.5%) | 5 (10.8%) | 0.244 |
| IHD, n (%) | 7 (17.5%) | 12 (26.0%) | 0.486 |
| Atrial fibrillation, n (%) | 2 (5%) | 8 (17.4%) | 0.147 |
| Hyperlipidemia, n (%) | 15 (37.5%) | 17 (36.9%) | 1.000 |
| Total cholesterol, mean (sd) mmol/l | 5.26 (1.23) (n=35) | 5.02 (1.14) (n=41) | 0.382 |
| Positive family history of stroke, n (%) | 5 (12.5%) | 3 (6.5%) (n=45) | 0.584 |
| **Inflammation** |  |  |  |
| CRP, median (Q1 – Q3) mg/L | 1.36 (0.74 – 3.10) | 1.68 (0.84 – 3.25) | 0.703 |
| TNF, median (Q1 – Q3) pg/mL | 0.92 (0.72 – 1.37) | 0.85 (0.76 – 1.37) | 0.849 |
| IL-6, median (Q1 – Q3) pg/mL | 2.57 (1.77 – 4.26) | 2.54 (1.81 – 3.57) | 0.962 |
| **Endothelial dysfunction** |  |  |  |
| ICAM, mean (SD) ng/mL | 155.03 (55.66) | 158.93 (45.39) (n=44) | 0.727 |
| vWF, mean (SD) iu/dL | 131.8 (43.72) | 133.9 (42.1) | 0.822 |
| **Thrombosis** |  |  |  |
| Fibrinogen, mean (SD) g/L | 3.70 (0.59) | 3.84 (0.61) (n=45) | 0.478 |
| t-PA, mean (SD) ng/mL | 6.94 (3.15) | 8.29 (2.87) | 0.042 * |
| D-dimer, median (Q1 – Q3) ng/mL | 91.5 (69 - 152) | 119.5 (71 – 165) | 0.828 |
| * p<0.05. **p<0.01. t-PA: tissue plasminogen activator; ICAM: inter-cellular adhesion molecule; vWF: von Willebrand factor; CRP: C-reactive protein; TNF: tumour necrosis factor-alpha; IL-6: interleukin-6. | | | |

***Supplementary Table 6. Association of t-PA with lacunar stroke subtype in non-smokers (n=86)***

|  | Regression coefficient (95% CI) | p value |
| --- | --- | --- |
| Lacunar stroke subtype | -1.372 (-2.837 to 0.093) | 0.066 |
| Age | -0.012 (-0.076 to 0.051) | 0.697 |
| Male sex | 0.155 (-1.405 to 1.717) | 0.843 |
| Hypertension | 0.454 (-1.015 to 1.924) | 0.540 |
| Diabetes | 0.122 (-1.777 to 2.022) | 0.898 |

***Supplementary Table 7. Correlations between plasma biomarkers used in linear regressions (n=98)***

|  | **Correlation coefficient** | **95% CI** | **P value** |
| --- | --- | --- | --- |
| **Inflammation** |  |  |  |
| _log_CRP and _log_TNF | 0.39 | 0.21 to 0.55 | <0.000001 |
| _log_CRP and _log_IL-6 | 0.61 | 0.46 to 0.72 | <0.000001 |
| _log_TNF and _log_IL-6 | 0.51 | 0.34 to 0.64 | <0.000001 |
| **Endothelial dysfunction** |  |  |  |
| ICAM and vWF | 0.11 | -0.09 to 0.31 | 0.28 |
| **Thrombosis** |  |  |  |
| Fibrinogen and t-PA | 0.33 | 0.14 to 0.50 | <0.0001 |
| Fibrinogen and _log_D-dimer | 0.25 | 0.05 to 0.43 | 0.01 |
| t-PA and _log_D-dimer | 0.09 | -0.11 to 0.29 | 0.36 |

***Supplementary Table 8. Explaining variance in visually rated WMH with different predictor variables (n=125)***

| Predictor variables | RSE | R^2^ |
| --- | --- | --- |
| *Baseline model*  Age^‡^, male sex, hypertension, smoking* | 1.374 | 0.226 |
| *Model 1*  Age^‡^, male sex, hypertension, smoking* + *Inflammation* | ***1.356*** | ***0.236*** |
| *Model 2*  Age^‡^, male sex, hypertension*, smoking* + *Endothelial activation* | ***1.368*** | ***0.234*** |
| *Model 3*  Age^‡^, male sex, hypertension, smoking* + *Thrombosis* | 1.410 | 0.213 |
| *Model 4*  Age^‡^, male sex, hypertension, smoking* + *Inflammation* + *Endothelial activation* + *Thrombosis* | ***1.351*** | ***0.238*** |
| * p<0.05, ^‡^ p<0.001.  *Inflammation* = logCRP, logTNF, logIL-6  *Endothelial activation* = vWF, ICAM  *Thrombosis* = t-PA, logD-dimer, fibrinogen  RSE = residual standard error (***bold*** = improvement over baseline, ie, reduction in RSE)  R^2^ = adjusted R-squared (***bold*** = improvement over baseline, ie, increase in R^2^) | | |
